# Demographic and clinical risk factors associated with hospital mortality after isolated severe traumatic brain injury: a cohort study

**DOI:** 10.1186/s40560-015-0113-4

**Published:** 2015-11-10

**Authors:** Vijay Krishnamoorthy, Monica S. Vavilala, Brianna Mills, Ali Rowhani-Rahbar

**Affiliations:** Department of Anesthesiology and Pain Medicine, University of Washington, 1959 NE Pacific Street, BB-1469, Seattle, WA 98195 USA; Department of Epidemiology, University of Washington, 1959 NE Pacific Street, F-250, Seattle, WA 98195 USA; Harborview Injury Prevention and Research Center, University of Washington, 1959 NE Pacific Street, BB-1469, Seattle, WA 98195 USA

**Keywords:** Trauma, Brain injury, Outcomes

## Abstract

**Background:**

Traumatic brain injury (TBI) is a major public health problem and a leading cause of death worldwide. A paucity of literature exists on risk factors for mortality in isolated severe TBI, a condition that is distinct from severe TBI in the setting of multisystem trauma. We determined risk factors for in-hospital mortality in this patient population.

**Methods:**

We conducted a retrospective cohort study using data from the National Trauma Databank from 2008–2012 to study all patients admitted with a diagnosis of severe TBI, excluding children, patients with non-isolated TBI, transfers, and hospitalization <48 h. We used multivariable Poisson regression to analyze the association between demographic, clinical, and facility-level characteristics and in-hospital mortality.

**Results:**

A total of 41,590 patients were included in our analysis. The cumulative incidence of in-hospital mortality was 10.2 %. In multivariable analysis, older age (RR 3.92, 95 % CI 3.54–4.34), male gender (RR 1.17, 95 % CI 1.09–1.25), admission hypotension (RR 1.83, 95 % CI 1.61–2.09), the need for mechanical ventilation (RR 4.18, 95 % CI 3.64–4.80), higher injury severity score (RR 1.86, 95 % CI 1.41–2.45), and poor initial neurologic grade (RR 3.06, 95 % CI 2.74–3.43) were associated with a higher risk for mortality.

**Conclusions:**

Admission hypotension and the need for mechanical ventilation were possible modifiable risk factors associated with increased in-hospital mortality following isolated severe TBI. Although risk factors for mortality are similar in isolated and non-isolated TBI, the underlying etiologies for hypotension and respiratory failure are likely different in both conditions and require further exploration.

## Background

Traumatic brain injury (TBI) is a major public health problem, affecting more than 1.7 million individuals annually in the USA alone [1]. Severe TBI, representing the greatest degree of injury, is associated with a high risk of mortality and functional disability. Although severe TBI is classically thought to occur in the setting of multisystem injury, current data suggest that the leading cause of TBI in the USA is falls [2], which may result in severe but isolated TBI. While prior studies have examined risk factors for morality in severe TBI in the setting of polytrauma [3], no large population-based investigation of mortality following isolated severe TBI has been conducted to date. This distinction is important because isolated severe TBI has distinct pathophysiology from TBI in the setting of multisystem trauma [4]; for example, early hypotension after multisystem trauma is generally secondary to hemorrhagic shock [5], while the etiology of hypotension after isolated TBI is less clear [6]. In addition, the largest of the prior studies of risk factors for mortality after severe TBI, based on the National Traumatic Coma Data Bank, used prospective data collected from patients with polytrauma and over a period between 1983 and 1988 [3], and advances have been made in TBI-specific surgical care as well as intensive care unit (ICU) care since this time period [7].

While increased clinical severity of TBI has been associated with a heightened risk of mortality, there is evidence from both the TBI and general critical care literature demonstrating that better hospital processes of care, such as adherence to guidelines, can contribute to decreased complications and improved outcomes in hospitalized patients [8–11]. Improved understanding of demographic and clinical risk factors for mortality after severe isolated TBI, in relationship with underlying hospital characteristics, may help improve processes of care and enhance risk stratification in this vulnerable patient population. The primary aims of our study were to: (1) describe the current epidemiology of severe isolated TBI in adults and (2) examine the association of demographic, clinical, and facility-level risk factors with in-hospital mortality in patients with isolated severe TBI.

## Methods

### Study design and population

We conducted a retrospective cohort study, using the National Trauma Data Bank (NTDB), a centralized national trauma registry created and operated by the American College of Surgeons. The NTDB represents the largest aggregation of United States trauma registry data ever assembled, with the mission to provide the trauma community with accessible and “consistent, quality data.” [12] The study did not require institutional review board approval, as the data were de-identified and did not meet the regulatory definition of human subjects research. We used NTDB data from 2008–2012, with linkage (using the de-identified patient number) of multiple data files (demographic, facility, admission, injury, complications, discharge diagnoses, and outcomes). We identified all admissions to participating trauma centers with severe isolated TBI. Because our goal was to examine mortality risk factors in the adult population, all patients less than 18 years of age at admission were excluded. We used the head abbreviated injury score (AIS) to classify TBI severity, as it modestly outperforms the Glasgow Coma Scale (GCS) as a predictor of long-term outcome after TBI [13]. We formed our TBI cohort using the injury file by keeping every patient with a head AIS score, and excluded any records with AIS scores for other body regions (other than head) to narrow the cohort to only isolated TBI patients. After this procedure, we excluded all patients with a head AIS score of less than 4, with represents the threshold for a severe injury [14]. We excluded patients with an AIS score of 6, as this score deems the injury as non-survivable. We also excluded patients admitted to the hospital for less than 48 h, based on the rationale that early mortality may not have been preventable. Lastly, we excluded all patients who were transferred, as we wanted to capture the admission clinical variables as close to the index injury as possible.

### Exposures, outcomes, and confounders

Our primary demographic and clinical exposures of interest were age, gender, the presence of hypotension at admission (defined as a systolic blood pressure <90 mmHg), admission heart rate, admission injury severity score (ISS), admission total Glasgow Coma Scale (GCS) score, and the need for mechanical ventilation during the hospitalization. Our primary facility-level exposures of interest were facility size (≤200 beds, 201–400 beds, >400 beds), teaching status (teaching versus non-teaching), and American College of Surgeons trauma level designation (trauma designated versus non-trauma designated facility). Our primary outcome was the occurrence of in-hospital mortality. Our analysis included all of the above variables, many of which are included in the “minimum set” of variables necessary for adjustment in mortality analyses using NTDB data, as previously reported [15]. In addition to our pre-specified variables included in our primary model (model 1), we created a second model (model 2), which attempted to capture in any further potential confounding factors or differences in processes of care among facilities. In addition to the variables in model 1, we also considered the following variables in model 2: race, insurance status, hospital profit status, number of neurosurgeons at the facility, and facility region.

### Statistical analysis

We described the demographic and clinical characteristics of our entire patient cohort. Continuous variables are reported as means, standard deviation, and range; and categorical variables are reported as counts and percentages. We calculated the cumulative incidence of in-hospital mortality (and 95 % confidence intervals), categorized by demographic, clinical, and facility-level exposure groups.

We calculated univariate and multivariable estimates of the association between demographic, clinical, and facility-level characteristics and in-hospital mortality using two Poisson regression models with clustered robust sandwich standard error estimates relaxing the assumption that observations from the same hospital are independent. In our model 1, we included age, admission heart rate, ISS score, total GCS score, and hospital size as categorical variables; and gender, the presence of hypotension at admission, the need for mechanical ventilation, trauma hospital designation, and hospital teaching status as binary variables. In model 2, we added race, insurance status, hospital profit status, number of neurosurgeons at the facility, and facility region as categorical variables. To test the robustness of our modeled covariates, we conducted a sensitivity analysis using continuous variables instead of categorical (for age, heart rate, ISS score, and GCS score). To test our assumption that hospitalization for at least 48 h was necessary for a facility characteristic to have an impact on mortality, we conducted sensitivity analyses restricting the duration of hospitalization of the population to >72 and >96 h. Because some covariates carried up to a 25 % missing proportion, we also performed a sensitivity analysis using multiple imputation by chained equations; this procedure was carried out separately for model 1 and model 2. We report effect measures as relative risk with 95 % confidence intervals. All analyses were conducted using Stata 13.0 (College Station, Texas).

## Results

We initially ascertained all severe isolated TBI patients from the 2008–2012 NTDB (*n* = 118,152). We excluded patients with an AIS score of 6 (*n* = 219) and patients who were admitted to the hospital for less than 48 h (*n* = 42,444). We excluded all patients who were either transferred into or out of their facility (*n* = 33,899). Our final cohort included 41,590 patients.

### Demographic and clinical patient characteristics

Demographic characteristics of our patient cohort included age, gender, race, and socioeconomic data (Table 1). The mean age of our sample was over 60 years old (61.1 ± 21.3 years), and patients were predominantly male (64 %). The majority of the patients in our sample were white (41.2 %), with a smaller proportion of African-American and Asian patients (6.3 and 1.8 %, respectively). A majority of patients in our sample had public insurance (51.9 %) rather than private insurance (20.5 %). Falls were the predominant mechanism of injury accounting for severe isolated TBI in our cohort (71.0 %).

**Table 1:** Demographic and clinical characteristics of isolated severe TBI patient cohort

	*N* = 41,590^a^
Age [mean ± SD, (range)]	61.1 ± 21.3 (18–110)
Male [*n*(%)]	26,604 (64.0 %)
Race [*n*(%)]	
White	17,135 (41.2 %)
African-American	2625 (6.3 %)
Asian/PI	749 (1.8 %)
Other	2527 (6.1 %)
Missing	18,554 (44.6 %)
Insurance [*n*(%)]	
Private	8516 (20.5 %)
Public	21,564 (51.9 %)
Self-pay	4427 (10.6 %)
Other	2749 (6.6 %)
Missing	4334 (10.4 %)
Admission systolic blood pressure [mean ± SD, (range)]	146.9 ± 29.4 (12–299)
Injury severity score [mean ± SD, (range)]	15.9 ± 6.3 (1–75)
Admission Glasgow Coma Scale (mean ± SD, range)^b^	12.6 ± 3.8 (3–15)
Admission heart rate [mean ± SD, (range)]	85.5 ± 20.9 (0–260)
Need for a ventilator [*n*(%)]	10,239 (24.6 %)
Injury mechanism [*n*(%)]^c^	
Motor vehicle-related	3896 (9.4 %)
Fall	29,512 (71.0 %)
Firearm	1399 (3.4 %)
Transport, other	973 (2.3 %)
Struck by/against	3176 (7.6 %)
Other, specified and classifiable	216 (0.5 %)
Other	2421 (5.8 %)

^a^May not reflect denominator for all characteristics, due to missing data

^b^TBI severity determined by head AIS score (see Methods)

^c^Slightly greater than total because three patients had multiple codes for “primary mechanism”

Initial physiologic characteristics of our patient cohort included measures of cardiac and pulmonary function, injury severity, and degree of neurologic compromise (Table 1). The mean systolic blood pressure (147 mmHg) and heart rate (86 bpm) were within the normal physiologic range, although substantial variation existed in our sample as evidenced by a large range of systolic blood pressures (12–299 mmHg) and heart rates (0–260 bpm). The mean injury severity of our cohort was high (ISS score 15.9), although the clinical neurologic severity appeared only moderate (mean GCS 12.6). Almost one-fourth (24.6 %) of the patients in our sample either arrived at the hospital intubated or developed respiratory failure requiring intubation and mechanical ventilation during hospitalization.

### Mortality experience after isolated severe TBI

In-hospital mortality occurred in 4228 patients admitted for longer than 48 h, giving a cumulative mortality of 10.2 %. Cumulative mortality stratified by demographic, clinical, and facility-level characteristics is shown in Table 2. The proportion of patients who experienced in-hospital mortality was higher among older patients (12.6 versus 7.5 % when comparing patients >80 years old to patients 18–44 years old). Greater injury severity was associated with greater levels of mortality (13 versus 3.3 %, comparing patients with ISS < 9 to patients with ISS > 16), as was worse initial neurologic grade (34.7 versus 5.5 % when comparing patients with a GCS ≤ 8 to patients with a GCS ≥ 13). Patients with hypotension at admission experienced higher levels of mortality (30.2 versus 10.5 %), as did patients with respiratory failure requiring intubation and mechanical ventilation (28.6 versus 4.1 %). Patients at university/teaching hospitals experienced a higher cumulative mortality (11.3 versus 9.4 %), as did patients at large hospitals (10.5 versus 8.7 % at small hospitals). Mortality experience was similar at trauma-designated and non-trauma hospitals (10.2 and 10.3 %, respectively).

**Table 2:** Demographic, clinical, and facility characteristics and cumulative mortality

Exposure	Total patients	Number died	Cumulative mortality (%)	95 % CI
Demographic characteristics				
Age				
18–44	9832	766	7.80	7.3–8.3 %
45–64	10,749	994	9.20	8.7–9.8 %
65–79	10,460	1137	10.90	10.3–11.5 %
80+	10,549	1331	12.60	12.0–13.3 %
Gender				
Male	26,604	2856	10.70	10.4–11.1 %
Female	14,927	1371	9.20	8.7–9.7 %
Clinical characteristics				
Cardiovascular				
Admission hypotension (SBP <90 mmHg)	524	158	30.20	26.2–34.3 %
Admission normotension (SBP ≥90 mmHg)	32,700	3437	10.50	10.2–10.8 %
Admission heart rate <60 bpm	2184	352	16.10	14.6–17.7 %
Admission heart rate 60–100 bpm	24,790	2401	9.70	9.3–10.1 %
Admission heart rate >100 bpm	6407	883	13.80	12.9–14.7 %
Respiratory				
Need for mechanical ventilation during hospitalization	10,239	2927	28.60	27.7–29.5 %
No need for mechanical ventilation during hospitalization	31,351	1301	4.10	3.9–4.4 %
Injury severity				
ISS <9	1330	44	3.30	2.4–4.4 %
ISS 9–15	11,476	431	3.80	3.4–4.1 %
ISS 16+	26,704	3481	13.00	12.6–13.4 %
Neurologic				
GCS 13–15	23,346	1276	5.50	5.2–5.8 %
GCS 9–12	3156	449	14.20	13.0–15.5 %
GCS ≤8	5096	1770	34.70	33.4–36.1 %
Facility characteristics				
Teaching status				
Community/non-teaching hospital	23,985	2246	9.40	9.0–9.7 %
University/teaching hospital	17,605	1982	11.30	10.8–11.7 %
Hospital bed size				
Small (<200 beds)	2139	187	8.70	7.6–10.0
Medium (201–400 beds)	12,382	1188	9.60	9.1–10.1 %
Large (>400 beds)	27,069	2853	10.50	10.2–10.9 %
Trauma hospital designation				
Trauma hospital	26,006	2648	10.20	9.9–10.6 %
Non-trauma hospital	14,778	1529	10.30	9.9–10.8 %

All total patient columns do not add up to the same value due to missing data

### Demographic, clinical, and facility-level risk factors for mortality

The association between demographic, clinical, and facility-level characteristics and in-hospital mortality is shown in Table 3. In multivariable analysis using our primary variables of interest (model 1), older age (RR 3.92, 95 % CI 3.54–4.34), male gender (RR 1.17, 95 % CI 1.09–1.25), admission hypotension (RR 1.83, 95 % CI 1.61–2.09), the need for mechanical ventilation (RR 4.18, 95 % CI 3.64–4.80), high injury severity (RR 1.86, 95 % CI 1.41–2.45), and poor initial neurologic grade (RR 3.06, 95 % CI 2.74–3.43) were associated with greater in-hospital mortality. With the addition of further possible confounding process of care variables to our initial multivariable model (model 2), all of the above factors remained significant. Multiple sensitivity analyses using continuous rather than categorical physiologic variables, testing longer duration of hospitalization (>72 and >96 h), and using multiple imputation by chained equations to account for missing data did not result in a meaningful change in effect measures or statistical significance for all variables except for gender, which lost significance when duration of hospitalization was set at >96 h.

**Table 3:** Association between demographic, clinical, and facility characteristics and in-hospital mortality

Exposure	Model 1 (RR, 95 % CI)^a^	Model 2 (RR, 95 % CI)^b^
Demographic characteristics		
Age		
18–44	ref	ref
45–64	1.63 (1.49–1.79)	1.62 (1.40–1.89)
65–79	2.58 (2.34–2.85)	2.63 (2.23–3.10)
80+	3.92 (3.54–4.34)	3.84 (3.22–4.58)
Male gender	1.17 (1.09–1.25)	1.12 (1.01–1.25)
Clinical characteristics		
Admission hypotension (SBP <90 mmHg)	1.83 (1.61–2.09)	1.71 (1.37–2.13)
Admission heart rate <60 bpm	ref	ref
Admission heart rate 60–100 bpm	0.82 (0.74–0.90)	0.88 (0.75–1.03)
Admission heart rate >100 bpm	0.88 (0.79–0.97)	0.95 (0.80–1.12)
Need for mechanical ventilation during hospitalization	4.18 (3.64–4.80)	4.40 (3.72–5.21)
Injury severity		
ISS <9	ref	ref
ISS 9–15	0.89 (0.67–1.17)	1.33 (0.75–2.37)
ISS 16+	1.86 (1.41–2.45)	2.92 (1.67–5.12)
Neurologic		
GCS 13–15	ref	ref
GCS 9–12	1.70 (1.51–1.90)	1.70 (1.43–2.02)
GCS ≤8	3.06 (2.74–3.43)	3.04 (2.60–3.55)
Facility characteristics		
University/teaching hospital	1.04 (0.95–1.15)	1.04 (0.91–1.20)
Hospital bed size		
Small (<200 beds)	ref	ref
Medium (201–400 beds)	1.00 (0.82–1.23)	1.11 (0.87–1.41)
Large (>400 beds)	1.04 (0.95–1.15)	1.18 (0.92–1.50)
Trauma hospital designation	0.99 (0.90–1.09)	0.95 (0.84–1.08)

^a^Model 1 includes trauma designation, teaching status, hospital size, age, gender, admission hypotension, admission GCS, admission ISS, and ventilator requirement during hospitalization

^b^Model 2 includes all variables in model 1 as well as race, insurance status, hospital profit status, number of neurosurgeons at facility, and facility region

## Discussion

The findings from our study demonstrated that among patients with isolated severe TBI hospitalized for greater than 48 h, falls were the predominant mechanism of injury, in-hospital mortality was high (cumulative incidence of 10.2 %), and older age, male gender, high injury and neurologic severity, and poor hemodynamic and respiratory function are associated with a higher incidence of in-hospital mortality. Thus, initial physiologic severity represents one of the most important risk factors for mortality, and this association remained robust even after adjusting for factors that may represent differences in crude measures of processes of care. While this data is similar to risk factors for mortality in TBI in the setting of multisystem trauma, the underlying etiologies of physiologic dysfunction, especially hypotension, are likely different.

There are no prior large epidemiologic investigations of risk factors for mortality in isolated severe TBI patients. Several smaller studies have explored risk factors for mortality in TBI in the setting of multisystem trauma. In an analysis of 734 patients with severe TBI in the setting of polytrauma from the National Traumatic Coma Databank, Piek and colleagues [3] documented that shock (systolic blood pressure ≤90 mmHg for greater than 30 min) and pulmonary infections were both significant independent predictors for an unfavorable outcome after severe TBI. Corral and colleagues [16] studied 224 patients retrospectively with severe TBI (the majority in the setting of polytrauma) and reported that the development of ICU hypotension and respiratory failure were both common (44 and 41 % of patients, respectively)—while they were both increased in hospital length of stay and morbidity, only hypotension in the most severe patients (GCS 3–5) increased mortality. In a sample of 373 patients with a variety severe neurologic disorders, Mascia and colleagues [17] found that, in addition to poor GCS scores, both cardiovascular and respiratory failure were independently associated with a higher risk of ICU mortality. In a prospective study of 134 patients (of which only 38 % had an isolated TBI), Zygun and colleagues [18] documented an incidence of ventilator-associated pneumonia of 45 %—while not associated with increased mortality, respiratory complications increased both hospital length of stay and morbidity. Lastly, in the placebo arm of a randomized controlled trial of low dose steroids for the prevention of hospital-acquired pneumonia among patients with severe TBI (168 patients, of which only 2.3 % had isolated TBI), Asehnoune and colleagues [19] documented an ICU mortality of 12 %. Thus, the overall mortality experience in severe TBI patients with and without multisystem trauma appears to be similar. Furthermore, while the mechanisms of hypotension and respiratory failure may differ in TBI patients with and without multisystem trauma, their effects on mortality in both disease paradigms are generally consistent.

To our knowledge, no prior studies have examined the relationship of facility-level characteristics and in-hospital mortality in the adult isolated TBI population, although this effect has been shown in other adult disease paradigms and in pediatric TBI. Allareddy and colleagues [20] showed that among 22,932,948 hospitalizations for major surgical procedures, admission to a larger hospital was associated with a reduction in risk of development of methicillin-resistant *Staphylococcus aureus* infections. Brown and colleagues [21] demonstrated that among 118,611 Medicare beneficiaries hospitalized for acute myocardial infarction, larger hospitals had a significantly reduced 30-day hospital readmission rate, compared to smaller hospitals. In the pediatric TBI population, it has been shown that hospitals with improved guideline adherence have reduced mortality and improved functional outcomes [11]; in addition, regional variation in mortality after pediatric TBI has been demonstrated as well [22]. In our study, we pre-specified facility-level factors in an attempt to adjust for differences in processes of care across facilities, but our hospital characteristics may have only served as a crude proxy and likely did not capture with enough granularity factors such as adherence to guidelines and evidence-based care pathways. Furthermore, facility characteristics may plausibly affect mortality after TBI in the setting of polytrauma, which often requires more complex care, although this requires further investigation. In addition, future research must continue to examine processes of care in adult TBI with improved granularity.

While the risk factors of older age, male gender, and initial injury and neurologic severity are likely non-modifiable, hypotension and respiratory failure may potentially be correctable, or even preventable, after isolated severe TBI. While hypotension following TBI in the setting of multisystem trauma is often the result of hypovolemia and blood loss from other injuries, hypotension after isolated TBI may involve other factors, including cardiac dysfunction [6, 23] and a maladaptive catecholamine excess state that may result in non-neurologic organ dysfunction [24]. Respiratory failure requiring mechanical ventilation may also be a potentially preventable complication after isolated severe TBI, particularly with regard to the prevention of hospital-acquired pneumonia, which increases the duration of mechanical ventilation and length of stay [18], increases medical costs [25], as well as increases secondary brain injuries such as fever and hypoxemia [26]. Patients with severe TBI may be at a particularly high risk of developing hospital-acquired pneumonia and respiratory failure due to impaired airway protective reflexes, dysphagia, and the subsequent risk of aspiration [27]. Regardless of the underlying cause, early and aggressive correction of hypotension and prevention of pneumonia after isolated severe TBI is likely warranted in order to minimize secondary brain injuries.

There are some limitations to our study. First, given our large retrospective data analysis, especially from a registry that was not collected for the purpose of research, certain variables may be prone to inaccuracies and coding errors. Some variables (especially admission GCS, hypotension, and heart rate) were prone to missing data approaching 25 %, although upon our investigation of the data, the missingness appeared to be at random when stratified by all exposure categories; furthermore, sensitivity analyses using multiple imputation revealed no meaningful changes to our risk estimates or confidence intervals. Second, while a great deal of diagnostic data is captured in the NTDB dataset, granular data, especially regarding physiologic variables, is not captured, thus, we are unable to capture the influence of ICU physiologic changes on outcomes. Third, while we a priori chose to base our TBI severity criteria on head AIS score, our resulting cohort may in fact have been more representative of moderate and severe injuries, as the mean GCS score was in the moderate range; despite this, the head AIS score both is a superior anatomic measure of injury severity and has been shown to be superior to GCS in prediction of long-term outcome after TBI [13]. Lastly, the necessary time for admission and facility characteristics to have a meaningful impact on outcomes has not been well described in the literature and the time period of 48 h seemed reasonable to us; despite this assumption, multiple sensitivity analyses using other time points revealed no significant changes to our risk estimates.

## Conclusions

There is a high burden of in-hospital mortality in patients with isolated severe TBI. While there are other non-modifiable risk factors with higher relative risks, admission hypotension and the need for mechanical ventilation are potentially modifiable risk factors that are strongly associated with mortality. Prevention and aggressive management of early hypotension and respiratory complications may help reduce mortality after isolated severe TBI. Although risk factors for mortality are similar in isolated and non-isolated TBI, the underlying etiologies for hypotension and respiratory failure are likely different in both conditions and requires further exploration in future studies.
